# The Integral Role of Radiology in the Diagnosis and Management of COVID-19–Associated Mucormycosis Infections

**DOI:** 10.4269/ajtmh.21-1135

**Published:** 2022-02-09

**Authors:** Anuj Prabhakar, Nidhi Prabhakar, Mandeep Garg, Ajay Kumar

**Affiliations:** Department of Radiodiagnosis & Imaging, Post Graduate Institute of Medical Education, Chandigarh, India

## Abstract

Recently, there has been a sudden surge in COVID-19–associated mucormycosis (CAM) infections. Rhino-oculo-cerebral and pulmonary mucormycosis are the two most common forms of CAM. Radiology plays an integral role in the management of CAM. Computed tomography (CT) determines gross bony and soft tissue involvement in COVID-19–associated rhino-oculo-cerebral mucormycosis, whereas magnetic resonance imaging helps in evaluation of the orbital and intracranial extension. Paranasal sinus soft tissue with extrasinus infiltration with or without bony destruction is suggestive of COVID-19–associated rhino-oculo-cerebral mucormycosis. High-resolution CT chest scan has shown to be helpful in the diagnosis of COVID-19–associated pulmonary mucormycosis. Consolidation and cavitation are the most common imaging features. Other CT abnormalities include the reverse-halo sign, pleural effusion, ground-glass opacities, pneumothorax, nodules, and pulmonary embolism. A high index of suspicion with appropriate imaging findings can lead to the early diagnosis of CAM and timely initiation of antifungal treatment and/or surgical debridement, which can be lifesaving.

## INTRODUCTION

COVID-19 has wreaked havoc on healthcare systems around the world. There was a sudden surge of COVID-19–associated mucormycosis cases, following the second wave of the COVID-19 pandemic. A systematic review published in August 2021 described 275 published cases of COVID-19–associated mucormycosis, out of which maximum have been reported in India (233), followed by Iran (19) and the United States (9). ^1^ Mucormycosis is an angioinvasive fungal disease with high fatality rates. Rhino-ocular/rhino-oculo-cerebral and lungs are the two most common locations affected by COVID-19 associated mucormycosis. Involvement of other areas such as the gastrointestinal system, bones, cutaneous, or disseminated disease has been less commonly reported. ^1^ COVID-19 afflicted or recovered patients presenting with symptoms and signs corresponding to the affected organ system should be carefully evaluated for the possibility of mucormycosis infection. ^2^ Rhino-oculo-cerebral involvement may manifest as nasal congestion, discharge, headache, facial pain, blackish discoloration of the nose, epistaxis, facial swelling, proptosis, sudden loss of vision, diplopia, and jaw pain. Patients with pulmonary mucormycosis may exhibit chest pain, hemoptysis, reoccurring fever, or worsening dyspnea. Associated diabetes mellitus or a history of glucocorticoid use are potential red flags. Although the clinical symptoms of COVID-19–associated mucormycosis has been documented, ^2^ we emphasize the vital role played by imaging in its early diagnosis and in delineating the extent of involvement, thereby helping in management. Early diagnosis is essential because timely initiation of antifungal treatment and surgical debridement can save lives.

## COVID-19–ASSOCIATED RHINO-OCULAR/RHINO-OCULO-CEREBRAL MUCORMYCOSIS

The diagnosis of COVID-19–associated rhino-oculo-cerebral mucormycosis is made by a combination of clinical and imaging parameters. Endoscopy with histopathology is required to confirm the diagnosis. Imaging is helpful in suggesting the diagnosis and delineates the extent, which is essential for surgical management of the patient. Both computed tomography (CT) and magnetic resonance imaging (MRI) contribute to the management. CT helps delineate the extent of bony and soft tissue involvement, whereas MRI shows the involvement of sinuses, orbits, cavernous sinuses, palate, skull base, and intracranial structures with more clarity. Noncontrast CT of paranasal sinuses is the initial investigation of choice to evaluate suspected cases of COVID-19 associated rhino-oculo-cerebral mucormycosis. In cases with suspected intracranial or orbital involvement, contrast enhanced MRI of the maxillofacial region will allow precise assessment of the soft tissue extension. The area of coverage should include the cavernous sinuses also. Brain MRI with and without contrast may be performed in patients with suspected intracranial spread. ^3^

**Figure 1.**
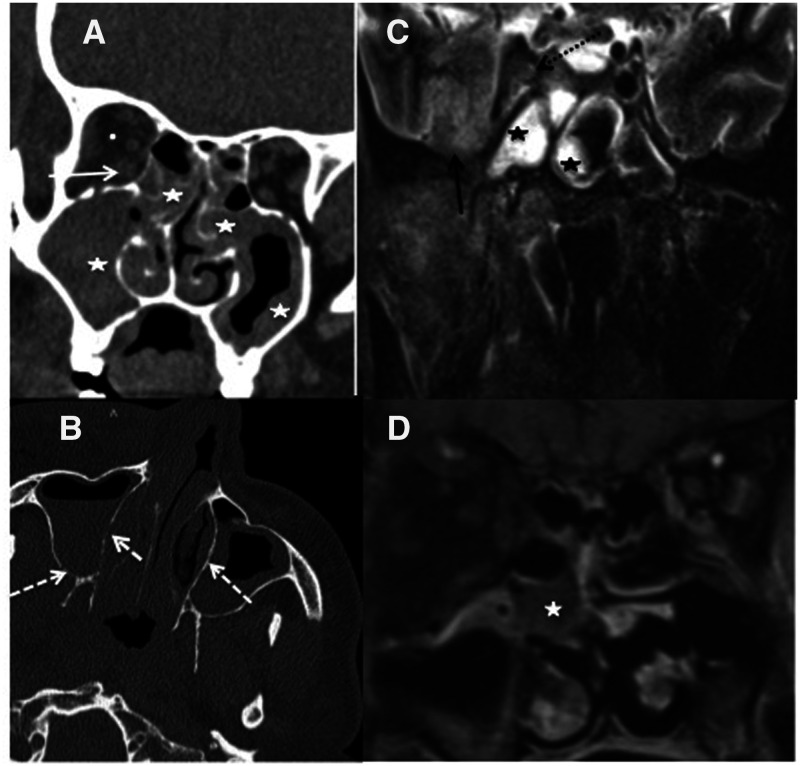
Radiological manifestations of proven cases of COVID-19–associated rhino-oculo-cerebral mucormycosis in different patients. (**A**) Computed tomography (CT) scan of coronal soft tissue window section showing mucosal thickening and soft tissue involving bilateral maxillary and ethmoid sinuses (white asterisks), fat stranding in the orbital fat (white dot), increase in bulk of extra ocular muscles with blurring of their margins (white arrow). (**B**) CT scan axial bony window section showing soft tissue in bilateral maxillary sinuses and right nasal cavity with presence of bony erosions in the walls of maxillary sinuses (white dashed arrows). (**C**) Magnetic resonance imagining (MRI) scan of coronal T2-weighted fat-saturated section showing soft tissue in sphenoid sinuses (black asterisks) with intracranial involvement shown by hyperintense signal in right temporal lobe (black arrow), ill-defined soft tissue in right cavernous sinus with occlusion of the right internal carotid artery (black dashed arrow). (**D**) MRI contrast-enhanced T1-weighted coronal section showing soft tissue thickening in right maxillary sinus with black turbinate sign (white asterisk).

COVID-19–associated rhino-oculo-cerebral mucormycosis shows initial involvement of nasal mucosa with soft tissue thickening. The ‘black turbinate’ sign is a distinctive feature, indicating acute invasive fungal sinusitis. It refers to nonenhancement of the nasal turbinates, due to infarction of overlying mucosa, on contrast-enhanced MRI of the paranasal sinuses. Bony erosions of the nasal septum and turbinates and partial or complete opacification of sinuses may be seen. Extrasinus spread may occur with or without associated bony destruction. There may be involvement of the buccal spaces, including the retromaxillary fat pad, the palate, or the oral cavity. The disease spreads into adjacent subcutaneous planes also. The presence of paranasal sinus involvement with extra sinus soft tissue infiltration is a characteristic finding that helps augment diagnostic accuracy (Figure 1). ^4^ Intracranial and intraorbital complications are most feared. Intraorbital involvement is seen most commonly as retroorbital fat stranding. There may be thickening of extraocular muscles, most commonly the medial rectus muscle. Careful evaluation for bony erosion of lamina papyracea and other orbital walls is needed. The optic nerve can be thickened or stretched. The disease process may extend to the orbital apex and involve the cavernous sinuses, which is seen as filling defects on contrast-enhanced CT or MRI. Dilatation of the superior ophthalmic vein with thrombosis may be seen. The intracranial extension may lead to complications such as leptomeningitis, cerebritis, or intracranial abscess formation. Basifrontal and temporal lobes are more commonly affected in such cases. ^4^ Vascular involvement may present as arteritis, pseudoaneurysm formation, or intraluminal thrombosis with secondary brain infarction. Post-gadolinium imaging helps in identifying these intracranial complications. Newer sequences such as susceptibility weighted imaging (SWI) and diffusion weighted imaging (DWI) play an important role in evaluation of intracranial complications. Hypointensity may be noted on SWI, surrounding the intraparenchymal lesions, due to micro-hemorrhages or mineral elements produced by invading fungi. DWI helps in identifying acute arterial territory infarcts. Also, a peripheral rim of restriction may be seen in fungal abscesses on DWI. ^5^^,^ ^6^

The differentiation of acute invasive fungal sinusitis from chronic sinusitis is essential. Chronic sinusitis presents as mucosal thickening or polyposis with sclerosis of underlying bones without evidence of bony destruction or erosions. ^4^ It is also challenging to differentiate aspergillosis from mucormycosis on imaging, but a few differentiating points have been reported. Aspergillosis more commonly has sinocranial involvement in the absence of orbital involvement, dural-based T2 hypointense masses on MRI, and unilateral (versus bilateral in mucormycosis) sinonasal cavity involvement. ^7^ On the other hand, thrombosis due to the involvement of vessel walls and a black turbinate sign are more common in mucormycosis infection. ^4^

Recent case series have been published describing the imaging findings of COVID-19–associated rhino-oculo-cerebral mucormycosis. In a study by Joshi et al., ^8^ 25 patients with COVID-19–associated rhino-oculo-cerebral mucormycosis were evaluated. They found 80% of patients with sinus wall destruction and 44% with air within the bony sinus walls. The maxillary sinus (100%) and ethmoid sinuses (76%) were most commonly involved; 28% had intracranial complications, and 36% had cavernous sinus involvement. Ten of these 25 patients underwent MRI, of which eight (80%) showed the black turbinate sign. In a study by Desai et al. ^9^ the maxillary sinus and the ethmoid sinuses were seen to be the most commonly involved (52% and 38% of patients, respectively); 36% had cavernous sinus involvement, 6% of patients had intracranial involvement, 70% orbital involvement, and 84% had bony erosions.

The largest reported series of rhino-oculo-cerebral mucormycosis, without COVID-19 (i.e., published before the COVID-19 pandemic), by Therakathu et al. ^10^ studied 43 patients and found that the ethmoid and maxillary sinuses were most commonly involved, in 86% and 79% of the patients, respectively. Orbital involvement was seen in 76% of patients, whereas intracranial involvement was seen in 31% of the patients and bony involvement in 40% of the patients. Fourteen percent showed cavernous sinus involvement. Postcontrast MRI was available in 14 patients, of which only two (0.14%) showed black turbinate sign. All the imaging studies on rhino-oculo-cerebral mucormycosis patients, whether COVID-19–associated or not, found maxillary and ethmoid sinuses to be the most commonly involved. There was bony involvement in 80% to 84% of patients of COVID-19–associated mucormycosis, whereas non-COVID-19–associated mucormycosis patients showed bony destruction in 40% of patients. No significant difference was seen on imaging, with respect to intracranial complications (varied from 6% to 31%), cavernous sinus involvement (14–36%), and orbital involvement (70–76%), in patients of rhino-oculo-cerebral mucormycosis, whether COVID-19–associated or not. However, all these studies involved a small number of patients (varying from 25 to 50); hence an accurate comparison can only be possible with the emergence of new data on larger cohorts of subjects.

## COVID-19–ASSOCIATED PULMONARY MUCORMYCOSIS

**Figure 2.**
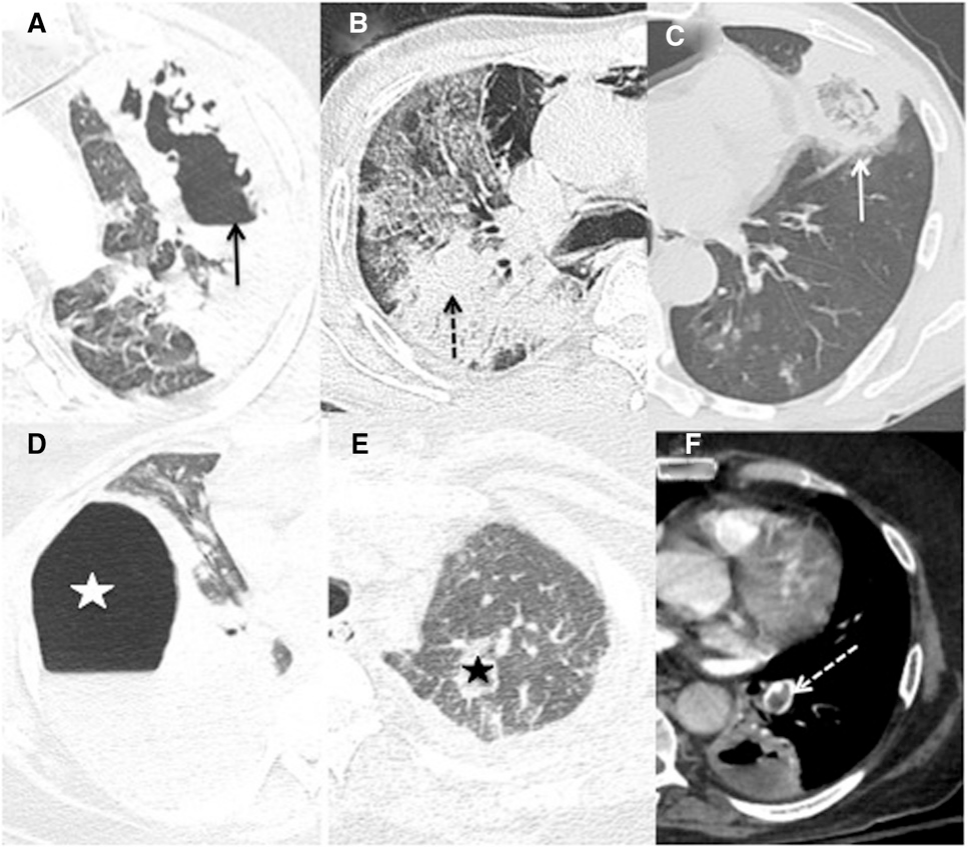
Spectrum of computed tomography (CT) chest findings in proven cases of COVID-19–associated pulmonary mucormycosis. (**A**) Cavitation (black arrow), (**B**) consolidation (black dashed arrow) with ground-glass opacities, (**C**) reverse-halo sign (white arrow), (**D**) hydropneumothorax (white asterisk), (**E**) nodule (black asterisk), and (**F**) pulmonary embolism (white dashed arrow).

Lungs are the other commonly involved organs after the rhino-oculo-cerebral system. COVID-19–associated pulmonary mucormycosis (CAPM) is more difficult to diagnose and has higher mortality than nonpulmonary mucormycosis. ^11^ High-resolution CT (HRCT) has been shown to be helpful in the diagnosis of COVID-19–associated pulmonary mucormycosis. A contrast-enhanced CT chest is ideal to rule out vascular complications of mucormycosis like thrombosis and pseudoaneurysm. ^12^ Garg et al. ^11^ evaluated the CT findings in 16 patients of CAPM and found consolidation (68.8 HRCT) and cavitation (68.8%) to be the most common imaging features. Other CT abnormalities included the reverse-halo sign (12.5%), pleural effusion (46.7%), ground-glass opacities (50%), pneumothorax (18.8%), nodules (18.8%), and pulmonary embolism (12.5%) (Figure 2). Hammer et al. ^12^ reported the chest CT findings in 30 patients of pulmonary mucormycosis before the COVID-19 pandemic. They found 67% patients showing the reverse-halo sign, 53% patients showing halo sign and 17% patients with multifocal pneumonia pattern. Choo et al. ^13^ studied the sequential changes on CT scan in pulmonary mucormycosis without COVID-19 and found that mucormycosis initially presents as a mass or masses with halo and reverse-halo sign on initial CT scan and later the ground-glass opacities decrease and internal necrosis and cavitation increase. One probable reason that cavitation has been found to be a more common finding than reverse-halo and halo sign in patients of CAPM is because these patients are being identified and diagnosed late in the course of the disease. ^11^ In addition, notable CT finding of pulmonary artery pseudoaneurysm has been reported in patients of pulmonary mucormycosis, both in cases with or without comorbid COVID-19. ^12^^,^ ^14^

Consolidation, ground-glass opacities, pleural effusion, pneumothorax, and pulmonary embolism can be seen on CT in patients with COVID-19 pneumonia; ^15^^–^ ^17^ however, the presence of cavitation, nodules, halo sign, air-crescent sign, and reverse-halo sign should alert clinicians to investigate for evidence of possible fungal infection. The differentiation of CAPM from COVID-19–associated pulmonary aspergillosis infection is challenging. The presence of peribronchial consolidations, bronchial-wall thickening, and centrilobular nodules favors the diagnosis of aspergillosis, whereas a reverse-halo sign, pleural effusion, and concurrent sinus infection favor CAPM. ^18^ Bacterial pneumonias caused by *Streptococcus pneumoniae*,* Hemophilus influenzae*,* Pseudomonas aeruginosa*,* Staphylococcus aureus*,* Klebsiella pneumoniae*, and* Mycobacterium tuberculosis* can also show cavitatory lesions. However, the concurrent presence of random nodules, halo sign, reverse-halo sign, or sinus infection will lead to the probable diagnosis of CAPM. Mucormycosis infection can be confirmed by the evaluation of endotracheal aspirate, sputum, or fine needle aspiration cytology. Imaging helps in the diagnosis of CAPM and can alert the radiologist and the treating clinician to plan further work-up for mycological evidence and institute antifungal therapy in appropriate clinical settings.

## CONCLUSION

A high index of clinical suspicion followed by appropriate radiological investigations is essential for the early diagnosis of COVID-19–associated mucormycosis and initiation of antifungal treatment or surgical debridement, both of which can be lifesaving.
